# Steroid Receptor RNA Activator Protein (SRAP): a potential new prognostic marker for estrogen receptor-positive/node-negative/younger breast cancer patients

**DOI:** 10.1186/bcr2359

**Published:** 2009-09-09

**Authors:** Yi Yan, George P Skliris, Carla Penner, Shilpa Chooniedass-Kothari, Charlton Cooper, Zoann Nugent, Anne Blanchard, Peter H Watson, Yvonne Myal, Leigh C Murphy, Etienne Leygue

**Affiliations:** 1 Manitoba Institute of Cell Biology, 675 McDermot Avenue, R3E0V9, Winnipeg, Manitoba, Canada; 2 Department of Biochemistry and Medical Genetics, University of Manitoba, 770 Bannatyne Avenue, R3E0W3, Winnipeg, Manitoba, Canada; 3 Department of Physiology, University of Manitoba, 770 Bannatyne Avenue, R3E0W3, Winnipeg, Manitoba, Canada; 4 BC Cancer Agency's Trev & Joyce Deeley Research Centre, 2410 Lee Avenue, V8R 6V5, Victoria, BC, Canada

## Abstract

**Introduction:**

The steroid receptor RNA activator is a functional RNA suspected to participate in the mechanisms underlying breast tumor progression. This RNA is also able to encode for a protein, Steroid Receptor RNA Activator Protein (SRAP), whose exact function remains to be determined. Our aim was to assess, in a large breast cancer cohort, whether levels of this protein could be associated with outcome or established clinical parameters.

**Methods:**

Following antibody validation, SRAP expression was assessed by tissue-microarray (TMA) analysis of 372 breast tumors. Clinical follow-up and parameters such as steroid receptor and node status were available for all the corresponding cases. Immunohistochemical scores were independently determined by three investigators and averaged. Statistical analyses were performed using standard univariate and multivariate tests.

**Results:**

SRAP levels were significantly (Mann-Whitney rank sum test, *P *< 0.05) higher in estrogen receptor-alpha positive (ER+, n = 271), in progesterone receptor positive (PR+, n = 257) and in older patients (age > 64 years, n = 182). When considering ER+ tumors, PR+ tumors, or younger patients (≤ 64 years), cases with high SRAP expression had a significantly (Mantel-Cox test, *P *< 0.05) worse breast cancer specific survival (BCSS) than those with low SRAP levels. SRAP also appeared as a very powerful indicator of poor prognostic for BCSS in the subset of ER+, node negative and young breast cancer patients (Cox regression analysis, n = 60, BCSS Hazard Ratio = 8.61, *P *< 0.006).

**Conclusions:**

Our data suggest that SRAP levels might provide additional information on potential risk of recurrence and negative outcome in a specific set of patients with otherwise good prognosis when considering only estrogen receptor and nodal status.

## Introduction

Breast cancer remains the second leading cause of cancer-related deaths in women worldwide and is one of the most frequently diagnosed cancers with an estimated 1,000,000 new cases detected each year worldwide [1]. Following diagnosis, several critical prognostic and predictive markers are assessed in order to determine, for each patient, the most appropriate treatment to be administered. Estrogen receptor (ER) status, progesterone receptor (PR) status, nodal status, tumor size, and grade of malignancy are the classical parameters used to date by clinicians to narrow down prognoses and weight treatment options [2]. More recently, human epidermal growth receptor (HER)-2, which is over-expressed in about 25% of breast cancers and is associated with a more aggressive disease and a poorer outcome, has also been used as a prognostic and predictive marker [3]. Recent approaches such as gene profiling and tissue micro-arrays (TMAs) have increased our ability not only to identify new potential markers, but also to rapidly test their potential validity [4,5]. The more such molecules are identified, the higher become the odds of finding the optimal combination of markers allowing the determination of an 'ideal' treatment for any given patient [6].

The steroid receptor RNA activator (SRA) was originally identified as a functional non-coding RNA increasing the transcriptional activity of ligand-bound steroid receptors [7]. It is currently believed that this action is mediated by the formation, at the promoter of target genes, of regulatory complexes containing steroid receptors, SRA, and both positive and negative protein regulators [8-10]. SRA RNA is over-expressed during breast, ovarian and uterine tumorigenesis and tumor progression [11-14]. It has therefore been suggested that by increasing the activity of the ER, SRA could participate in the mechanisms underlying these events [7,12].

It has now been confirmed that coding SRA transcripts co-exist in breast cancer cells, with the previously described non-coding transcripts [15-17]. The corresponding endogenous protein, steroid receptor RNA activator protein (SRAP), has been detected by western blot in multiple cell lines as well as in muscle and breast tissue [15,17-19]. It has been suggested that, as its RNA counter-part, the protein might also regulate the activity of estrogen and androgen receptors [10,17-19]. This hypothesis is further corroborated by the identification of the RNA helicase p68, a well-characterized regulator of ER activity [20], in nuclear complexes co-immunoprecipitating with endogenous SRAP [21].

Overall, accumulated data raise the intriguing possibility that SRAP levels could be associated with ER activity and/or expression, and could also potentially reflect on the response of breast cancer patients to endocrine therapy. It has recently been reported that the relative proportion of coding and non-coding SRA transcripts varies from one breast tumor to another and might characterize particular tumor subgroups [22]. Altogether, this suggests that SRAP expression could also differ between cases and potentially be a prognostic and/or predictive indicator in breast cancer. To address this issue, we have herein investigated the use of TMAs for the expression of SRAP in 372 cases with a wide range of clinical parameters.

## Materials and methods

### Cell culture

Hela and Michigan Cancer Foundation (MCF)-7 cells (Cedarlane Laboratories Ltd., Burlington, ON, Canada) cells were cultured in DMEM (Gibco, Grand Island, NY, USA) medium supplemented with 5% FBS (Cansera, Rexdale, ON, Canada), penicillin (100 units/ml), streptomycin (100 μg/ml) (Gibco, Grand Island, NY, USA), and 0.3% glucose. Cells were grown in a 37°C humidified incubator with 5% carbon dioxide. Cells were transfected with empty vector or plasmids containing either the full SRA coding sequence and leading to the production of a SRAP-V5 tagged protein [16], or a pSuper.retro-SRA construct expressing a SRA-Interfering RNA (SRA-RNAi) SRA-Interfering RNA sequence [15], as previously described [15,16].

### Western blot

Total proteins were extracted from cells pellets as previously outlined [17]. Frozen breast tumor sections were lysed and sonicated for 30 seconds in ice-cold Sodium-dodecyl-sulfate-Isolation-Buffer (SIB) containing 60 mM α-glycerophosphate, 1% SDS and a mini-protease inhibitor cocktail tablet (Boehringer Mannheim, Indianapolis, IN, USA) per 10 ml extraction buffer. Samples were then centrifuged at 13,000 g for 20 minutes at 4°C and stored at -20°C until use. Protein concentration was determined using BCA kit (Pierce Company, Rockford, IL, USA). Samples containing 75 μg of total protein were subsequently analyzed by western blot as described previously [17], using a primary anti-SRAP rabbit polyclonal antibody (cat # A300-743A, Bethyl Laboratories, Montgomery, TX, USA) raised against the C-terminal extremity of SRAP (peptide 180-237 aa). Signal detection and documentation was performed using the ChemiDoc imaging system (Bio-Rad, Mississauga, ON, Canada).

### Antibody neutralization experiments

For neutralization experiments, 2 μg of SRAP-180-237 aa blocking peptide (cat # BP300-743 Bethyl Laboratories, Montgomery, TX, USA) was pre-incubated with 1 μg of 743A antibody for two hours at room temperature. A peptide, corresponding to 69 to 89 aa from the Small Breast Epithelial Mucin [23], was used as a non-specific blocking peptide.

### Immunofluorescence

Hela cells cultured on cover-slips were transfected with control empty vector or plasmid expressing V5-Tagged-SRAP or SRA-RNAi (see above). Twenty-four hours post-transfection, coverslips were washed with PBS and cells fixed with 4% formaldehyde (Sigma, Oakville, ON, Canada) and 4% sucrose (Sigma, Oakville, ON, Canada) for 15 minutes at room temperature. Fixed cells were then rinsed with PBS and permeabilized with 0.25% Triton-X100 (Sigma, Oakville, ON, Canada) in PBS for five minutes. After rinsing with PBS twice, non-specific binding sites were blocked with 10% BSA (Sigma, Oakville, ON, Canada) in PBS for 30 minutes at 37°C. Cells were then incubated overnight at 4°C with 743A Anti-SRAP antibody diluted at 1/200 in 3% BSA/PBS. After washing with PBS for 15 minutes, cells were incubated with anti-rabbit secondary antibody-Cy3 conjugate (Jackson, West Grove, PA, USA) at a 1/1000 dilution in 3% BSA/PBS for one hour at room temperature. Cell nuclei were stained with 1 μg/ml Hoechst (Invitrogen, Burlington, ON, Canada), and coverslips were mounted onto microscopy slides with FluorSave™Reagent (Calbiochem, La Jolla, CA, USA). Fluorescent images were captured and visualized with a Nikon Eclipse E1000 epifluorescent microscope at wavelengths of 552 to 620 nm (CY3), 440 to 450 nm (Hoechst) using ACT-1 software (Nikon, Mississauga, ON, Canada).

### Breast samples and tissue micro-arrays

All invasive breast cancer and normal breast samples used in the current study were obtained from the Manitoba Breast Tumor Bank, which operates with the approval of the Faculty of Medicine, University of Manitoba, Research Ethics Board [24]. The research reported in this manuscript has been performed with the approval of the Bannatyne Campus, University of Manitoba, Research Ethics Boards. As described previously, all tissues accrued to the bank from cases at multiple centers within Manitoba are frozen at -70°C immediately after surgical removal. A portion of the frozen tissue from each case is then processed to create matched formalin-fixed paraffin-embedded and frozen tissue blocks. The histopathology of all Manitoba Breast Tumor Bank cases has been previously assessed and entered into a computerized database to enable selection based on composition of the tissue as well as clinico-pathological parameters.

Matched frozen and paraffin-embedded sections corresponding to 20 invasive breast tumors and 6 reduction mammoplasties were first selected and evaluated for protein expression by western blot and immunohistochemistry, respectively. Tumor ER and PR levels (determined by ligand binding assay (LBA)) ranged from 2.3 to 180 fmol/mg protein and 4.5 to 42 fmol/mg protein, respectively. The age of patients ranged between 58 and 78 years, and tumor size varied from 15 to 50 mm.

TMAs corresponding to 450 ER positive (LBA > 3 fmol/mg total protein) and 255 ER negative (LBA ≤ 3 fmol/mg total protein) cases were constructed from primary invasive breast carcinomas, as described before [25,26]. Duplicate core tissue samples (0.6 mm diameter) were taken from selected areas of maximum cellularity for each tumor. Two controls, corresponding to cases positive and negative for SRAP expression, respectively, were included in each TMA run to check for run to run reproducibility. From our studies, cases with lost follow-up, unknown grade or nodal status, or data on SRAP expression (loss of a core during processing) were removed. All characteristics were ultimately available for 372 breast cases. Tumors corresponded to 271 cases associated with ER-positive status (ER > 3 fmol/mg total protein, as assessed by LBA) that were treated by surgery and then tamoxifen endocrine therapy with or without radiation therapy. Remaining tumors corresponded to 99 cases associated with ER-negative status (ER ≤ 3 fmol/mg total protein, as assessed by LBA) that were treated by surgery with or without radiation therapy, tamoxifen endocrine therapy or chemotherapy. The whole cohort has ER levels ranging 0 to 331 fmol/mg protein (median 23 fmol/mg protein) and spanned a wide range of PR levels 0 to 1591 fmol/mg protein (median 17.95 fmol/mg protein). Nottingham grade [27] was also known for all 372 tumors, which were assigned to low (n = 79, scores 3 to 5), moderate (n = 205, scores 6 to 7) or high (n = 88, scores 8 to 9) categories. Age of patients ranged from 25 to 92 years old (median 64 years). The clinical follow-up ranged from 1 to 179 months (median 85 months). Out of 372 cases, 172 had a recurrence of the disease and 141 died from the disease.

Breast tumor tissue sections and TMAs were stained with an anti-SRAP antibody (cat # A300-743A, Bethyl Laboratories, Montgomery, TX, USA) using an automated tissue immunostainer (Discovery Staining Module, Ventana Medical Systems, Tucson, AZ, USA) at a dilution of 1:250, as described previously [25,26]. Slides were viewed and scored using standard light microscopy.

### Quantification and SRAP staining cut-off selection

SRAP protein expression was assessed using a previously described semi-quantitative scoring consisting of an assessment of both staining intensity (scale 0 to 3) and the percentage of positive cells (0 to 100%), which, when multiplied, generate an H-score ranging from 0 to 300 [25,26]. TMA slides were independently scored by three investigators (GPS, CCP and YY). Average of values scored by the three investigators were calculated. There is no relevant clinical cut-off point reported for SRAP. The median H-score value of 76.67 was arbitrarily set as cut-point. Breast cancers were therefore considered high SRAP expressors when their average score exceeded 76.67 and low SRAP expressors when their H-score was lower than or equal to 76.67. Cohen kappa coefficient for the semi-quantitative H scores were 0.43, 0.50 and 0.65, indicating a moderate to substantial inter-rater agreement between the three readers [28].

### Statistical analysis

Differences between SRAP expression in different subgroups (such as ER positive versus ER negative or node positive versus node negative, for example) were tested using the Mann-Whitney rank sum test, two sided. Box and whiskers representation was performed with boxes at 25 to 75% and whiskers at 10 to 90%. Potential distributions of low and high SRAP cases in other clinical-pathological variables were tested using contingency methods (Fisher's exact test). Survival analyses were performed using the Mantel-Cox log-rank test to generate Kaplan-Meier curves. Breast Cancer Specific Survival (BCSS) was defined as the time from initial surgery to the date of death attributable to breast cancer only. Recurrence-free survival (RFS) was defined as the time from initial surgery to the date of clinically documented local or distant disease recurrence or death attributed to breast cancer. Deaths caused by other known diseases were censored. Statistical analyses were carried out using GraphPad Prism 5 (GraphPad, San Diego, CA, USA) and PASW Statistics 17 (SPSS Inc. Chicago, IL, USA).

Cox proportional hazards model was used to test the statistical independence and significance of the different predictors on BCSS (events modeled: deaths attributable to breast cancer only) and RFS (events modeled consisting of recurrences or deaths attributed to breast cancer). The predictors considered in the model were: age at diagnosis (age ≤ median age 64, age > median age 64); ER-alpha status (ER ≤ 3 considered negative, ER > 3 are positive); node status (positive, negative); SRAP (low SRAP ≤ 76.67, high SRAP > 76.67); all two, three and four-way interactions involving SRAP.

## Results

### Anti-SRAP antibody validation

**Figure 1:**
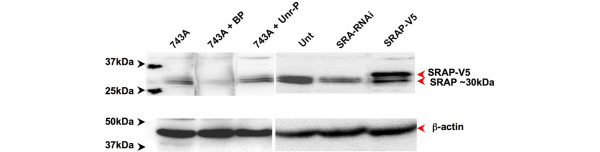
Western blot detection of 30 kDa SRAP in MCF-7 cells. Total proteins, extracted from MCF-7 breast cancer cells, were analyzed by western blot using 743A polyclonal anti-steroid receptor RNA activator protein (SRAP) antibody as described in the Materials and Methods section (743A). The antibody has been pre-incubated with its corresponding blocking peptide (743A + BP) or with an unrelated peptide (743A + Unr-P). In a parallel series of experiments, cells were transfected with an empty vector (Unt), SRA-RNAi or a construct encoding V5-tagged SRAP (SRAP-V5) as indicated in the Materials and Methods section. MCF = Michigan Cancer Foundation.

The ability of the 743A anti-SRAP antibody (Bethyl, Montgomery, TX, USA) to specifically recognize SRAP was first assessed by western blot analysis of total protein extracted from MCF-7 breast cancer cells, previously shown to express this protein [15-17]. A doublet, migrating at the expected apparent size of about 30 kDa, is specifically detected (Figure 1, left panel). As anticipated, the signal is decreased when the antibody is pre-incubated with an excess of corresponding blocking (743A + BP) but not unrelated (743A + Unr-P) peptide. The specificity of the signal recognized by 743A anti-SRAP antibody is further demonstrated by its decrease when cells are expressing SRA RNAi or by the appearance of an additional band in cells expressing a V5-Tagged-SRAP construct (Figure 1, right panel).

**Figure 2:**
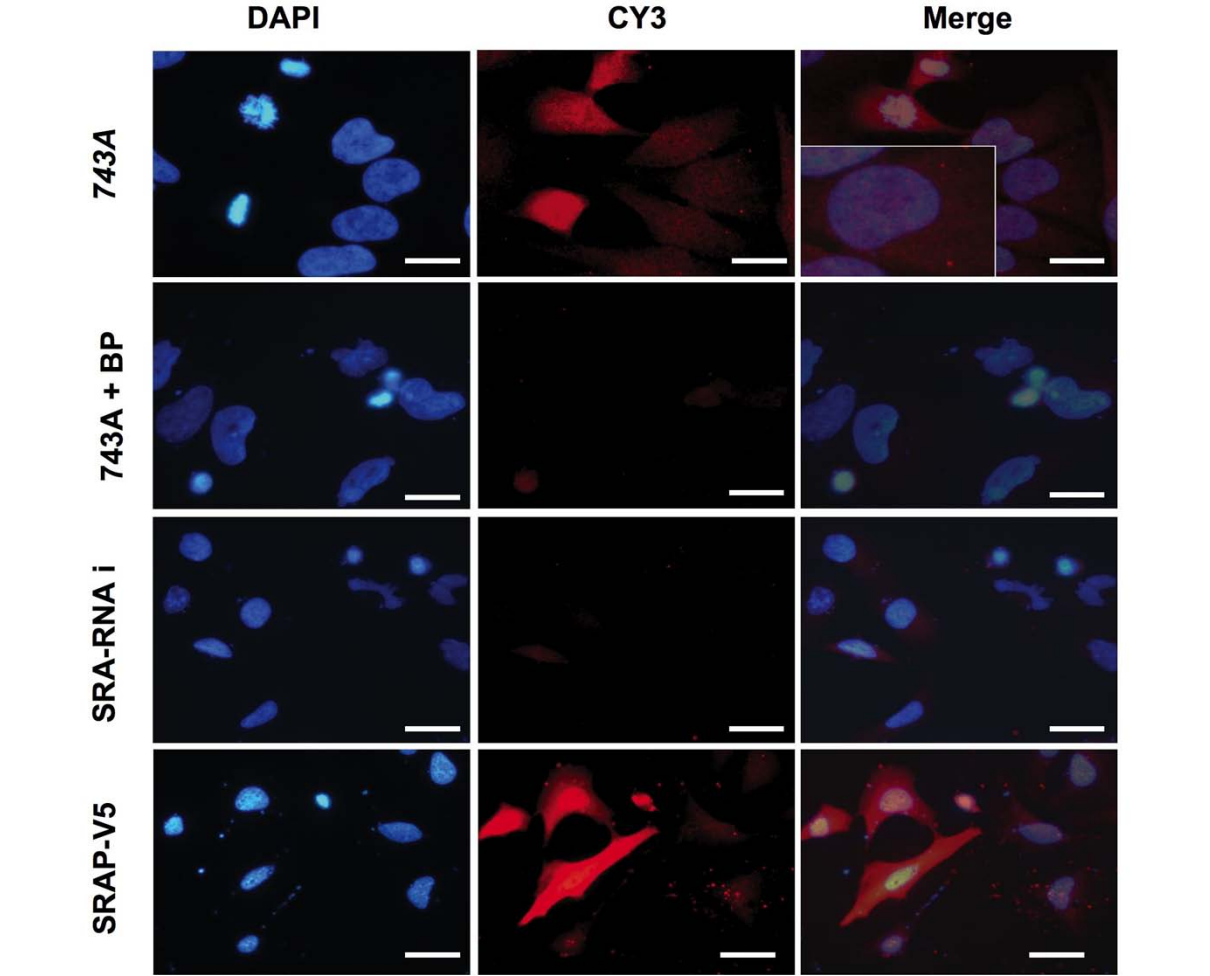
Detection of SRAP in Hela cells by fluorescent immunocytochemistry. Steroid receptor RNA activator protein (SRAP) was detected in untransfected (743A), steroid receptor RNA activator Interfering RNA transfected (SRA-RNAi) and SRAP-V5 infected Hela cells using 743A anti-SRAP antibody as described in Materials and Methods. 743A + BP = 743A antibody was pre-incubated in an excess of neutralizing peptide. Bar = 20 μm.

To further validate the use of 743A anti-SRAP antibody for *in situ *analyses, similar experiments were performed by immunofluorescence in Hela cells. As shown Figure 2, SRAP signal is detected in the nucleus and the cytoplasm of Hela cells (743A panels). As expected, this signal is decreased when the primary antibody is pre-incubated with the blocking peptide (743A + BP panels) or when cells expressed SRA RNAi (SRA-RNAi panel). Inversely, cells expressing exogenous V5-tagged-SRAP showed an increased signal detected with the 743A antibody (SRAP-V5 panels).

### Differential SRAP expression in breast tissues

**Figure 3:**
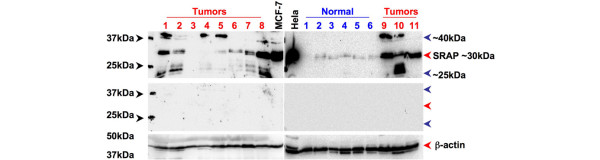
Western blot detection of 30 kDa, 25 kDA and 40 kDa SRAP in breast tumor tissues. Protein extracts from MCF-7 cells, Hela cells, breast tumors (1 to 9, red numbers) and normal (1 to 6 blue numbers) breast tissues were examined by western blot and visualized with 743A anti-steroid receptor RNA activator protein (SRAP) antibody (upper panel) as described in the Materials and Methods. The same extracts were analyzed with 743A anti-SRAP antibody pre-absorbed with an excess of blocking peptide (middle panel) or with an antibody targeting β-actin (lower panel). MCF = Michigan Cancer Foundation.

SRAP expression was subsequently assessed by western blot in a small series of 20 breast tumors and 6 normal breast samples as detailed in the Materials and Methods section. A signal, which varied in intensity between tumor samples and corresponding to SRAP of 30 kDa was detected in most cases (Figure 3, red arrow). Overall, this signal appears stronger in tumors than in normal tissues. It should however be emphasized that SRAP of 30 kDa is not detectable in all tumors (see Tumors 3 and 4) nor normal tissues (see Normal 1). Interestingly, two additional bands migrating at apparent sizes of 40 kDa (see Tumors 1, 9, 10) and 25 kDa (see Tumors 1, 2, 10) are also detected in some tumor cases but not in normal tissues. All these bands are specifically recognized by 743A anti-SRAP antibody, as shown by extinction of the signals in neutralization experiments (Figure 3, middle panel). It should also be noted, that in MCF-7 but not in Hela cells, overexposure of the blot led to the detection of the 40 kDa band.

**Figure 4:**
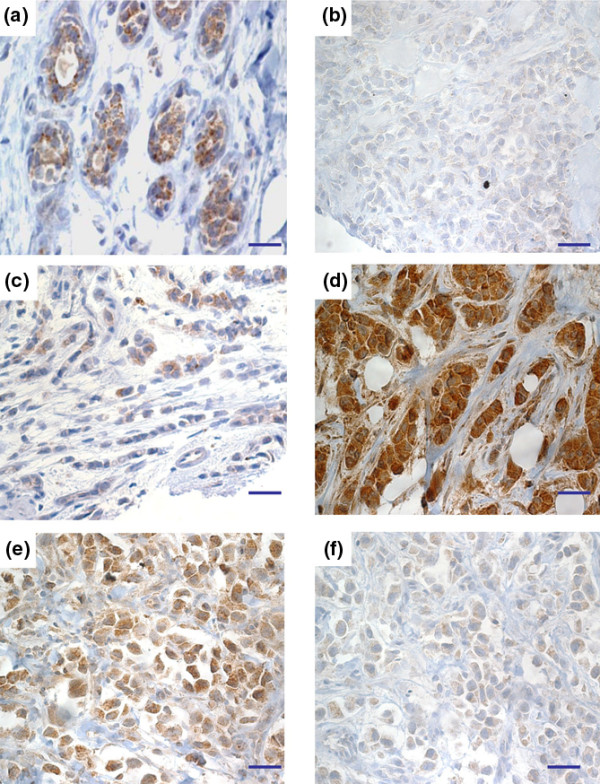
SRAP immunohistochemical detection in breast tissue sections. Steroid receptor RNA activator protein (SRAP) expression was assessed in **(a) **normal and **(b to f) **tumor paraffin-embedded tissue sections using 743A anti-SRAP antibody as described in the Materials and Methods section. **(f) **A serial section adjacent to section E has been treated with 743A pre-incubated with specific blocking peptide. Bar = 50 μm.

Immunohistochemical analysis performed on matched paraffin sections confirms that SRAP is detectable in epithelial cells within normal ducts (Figure 4a). A wide range of staining can be observed in breast cancers with some tumors showing a weak (Figures 4b, c) and others an intense staining (Figure 4d). The specificity of the staining is demonstrated by its decrease when the primary antibody is pre-incubated with the corresponding blocking peptide (Figures 4e, f) but not with a non-specific/irrelevant peptide (data not shown).

### Tissue micro-array analysis of SRAP expression in 372 breast cancer cases

**Figure 5:**
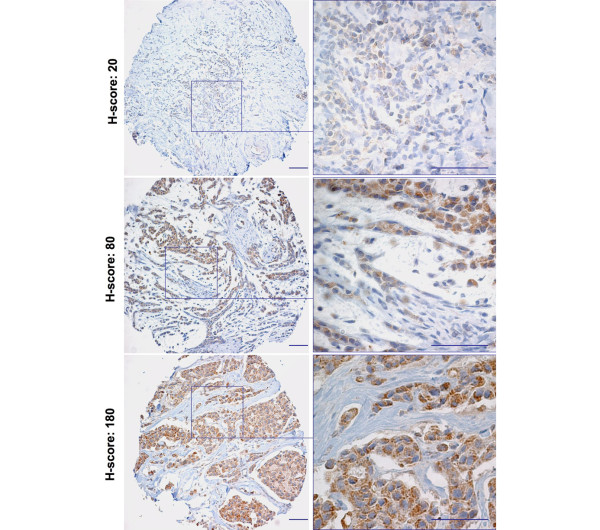
Tissue micro-array analysis of SRAP expression. Immunohistochemical detection of steroid receptor RNA activator protein (SRAP) was performed using 743A polyclonal anti-SRAP antibody on tissue micro-arrays corresponding to a large cohort of breast tumors. For each case, SRAP staining has been assessed and an H-score determined as detailed in the Material and Methods section. Examples of three cores with different H-scores are provided. Bar = 100 μm.

We have investigated SRAP expression in TMAs corresponding to a large cohort of breast cancer cases with different established clinical parameters. Staining and scoring were performed as described in the Materials and Methods section. Examples of staining and corresponding H-scores are shown in Figure 5. SRAP staining varies greatly from one sample to another, with H-scores ranging from 0 to 196.67 (n = 372, median = 76.67, average = 81.27). SRAP expression, ER/PR/node status, Nottingham grade [27], size of the tumor, patient age at surgery and clinical follow-up were available for 372 patients.

**Figure 6:**
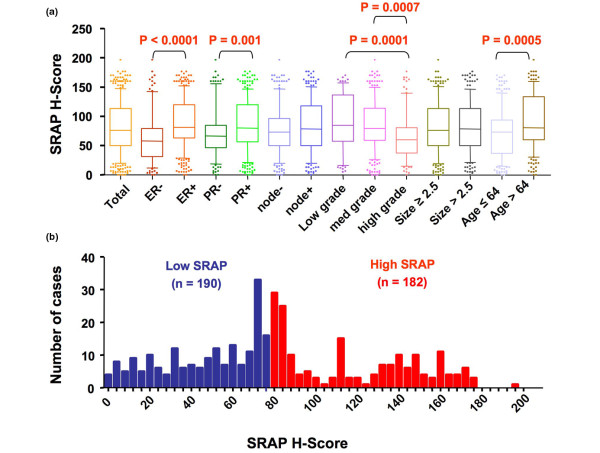
Distribution of SRAP H-scores in 372 breast cancer cases. **(a) **Box and whiskers representation showing the distribution of steroid receptor RNA activator protein (SRAP) H-scores in the whole cohort (Total) and in different subgroups. For each group the median is depicted by the thick horizontal bar, the box delineates 25^th ^to 75^th ^percentiles and the whiskers 10 to 90 percentiles **(b) **Frequency distribution of SRAP H-scores in the whole cohort (Total). Median H-score of 76.67 delineates Low SRAP and a High SRAP subgroups.

As illustrated in Figure 6a, significant (Mann-Whitney rank sum test, two-sided, *P *< 0.0001) H-score values are higher in ER-positive than in ER-negative tumors (ER positive, n = 271, median = 81.67 versus ER negative, n = 101, median = 58.33). Similarly, SRAP staining is significantly stronger in PR-positive than in PR-negative cases (PR positive, n = 256, median = 80 versus PR negative, n = 116, median = 66.67). Tumors with higher Nottingham grade had a significantly lower SRAP staining than both low and medium grade lesions (high grade, n = 88, median = 60 versus low grade, n = 79, median = 85 and medium grade, n = 205, median = 79.17). When cases were divided into two groups using the median age at surgery (median H-score 64), older patients had a significantly higher SRAP expression detected in their tumors than younger patients (older patients, n = 183, median = 80.83 versus younger patients, n = 189, median = 73.83). No difference in SRAP staining was observed between node-positive and node-negative tumors or between small and bigger lesions.

To further investigate potential non-random distributions of SRAP H-score staining in different tumor subgroups, we have arbitrarily divided the cohort into low and high SRAP expressors by selecting the median H-score value as cut-off point (low SRAP, H-score lower or equal to 76.67, n = 190; high SRAP, H-scores higher than 76.67, n = 182; Figure 6b). Contingency table analyses showed that low SRAP cases were significantly (Fisher's exact test) over-represented in ER-negative, PR-negative and higher grade tumors compared with ER-positive, PR-positive and lower grade tumors, respectively (Table 1). Similarly, a higher proportion of low SRAP expressors was found in lesions from younger patients. Identical proportions of low and high SRAP expressors were found in node-positive and node-negative tumors, and in small and large lesions.

**Table 1:** Distribution of low and high SRAP cases in case subgroups

Case subgroups	Low SRAP	High SRAP	Total	*P *value
	n (%)	n (%)		
**ER -**	72 (71)	29 (29)	101	**< 0.0001**
**ER+**	115 (42)	156 (58)	271	

**PR -**	78 (67)	37 (33)	115	**0.0053**
**PR+**	112(44)	145 (56)	257	

**Node -**	95 (55)	80 (45)	172	0.2545
**Node +**	95 (48)	102 (52)	200	

**Low (3-5)**	30 (39)	47 (61)	77	**< 0.0001**
**Med (6-7)**	97 (47)	109 (53)	206	
**High (8-9)**	63 (71)	26 (29)	89	

**Low size (≤ 2.5 cm)**	105 (54)	90 (46)	195	0.2992
**High size (> 2.5 cm)**	85 (48)	92 (52)	177	

**Age ≤ 64**	113 (60)	77 (40)	190	**0.0013**
**Age > 64**	77 (42)	105 (58)	182	

**Total**	190	182	372	

*P*-values correspond to Fisher's exact test. ER = estrogen receptor; PR = progesterone receptor; SRAP = steroid receptor RNA activator protein; + = positive; - = negative.

### Low SRAP expression is associated with longer survival among ER-positive, PR-positive and younger breast cancer patients

**Figure 7:**
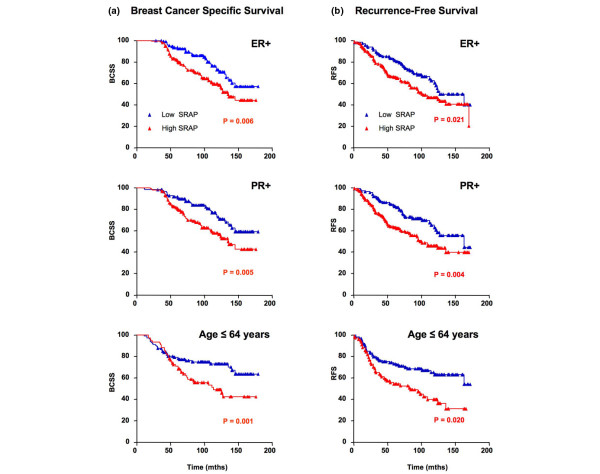
Low SRAP is an indicator of better survival for younger patients and patients with ER positive or PR positive tumors. Kaplan-Meier breast cancer-specific **(a) **death survival and **(b) **recurrence-free survival plots are shown for patients with ER+ tumors (n = 271), PR+ tumors (n = 256), and for patients younger than 65 years old (n = 189). *P *values correspond to Mantel-Cox log-rank test. ER = estrogen receptor; PR = progesterone receptor; SRAP = steroid receptor RNA activator protein; + = positive; - = negative.

When analyzing the cohort as a whole and most of the case subgroups detailed in Table 1, no difference was found in BCSS between high and low SRAP expressors (data not shown). Interestingly patients whose tumors were ER positive had a significantly longer BCSS and RFS (Figures 7a, b) when their primary tumor expressed lower levels of SRAP (Mantel-Cox, *P *= 0.0065 and *P *= 0.0212, respectively). When considering PR-positive cases, patients whose primary tumors expressed low SRAP also had a better BCSS and RFS (Figures 7a, b; Mantel-Cox, *P *= 0.0052 and *P *= 0.0041, respectively). In younger patients (age ≤ 64 years old, corresponding to values lower and equal to median age at surgery) Low SRAP levels were similarly associated with a longer BCSS and RFS (Figures 7a, b; Mantel-Cox, *P *= 0.0117 and *P *= 0.002, respectively). No significant differences between low SRAP and high SRAP were found in the whole cohort or any other subgroups defined in Table 1.

### Lower SRAP level is a significant predictor of survival in younger patients whose tumors are ER positive and node negative

**Figure 8:**
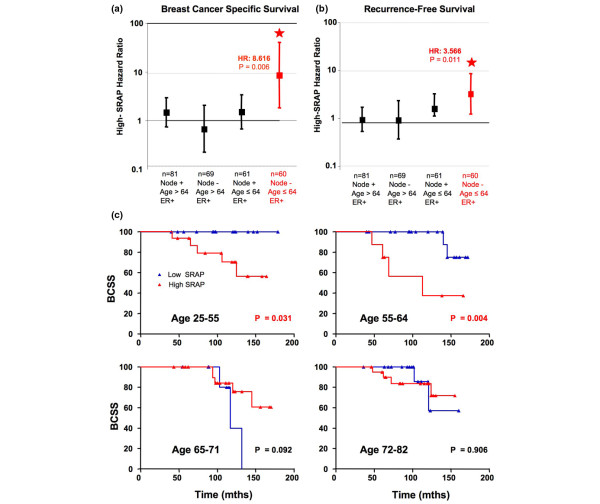
SRAP as a poor prognostic indicator in younger patients whose tumors are ER positive and node negative. Cox multivariate analyses have been performed as described in the Materials and Methods section. The events modeled were **(a) **death attributable to breast cancer, or **(b)** recurrence or death attributed to breast cancer. Hazard ratios within different estrogen receptor positive (ER+) subgroups are shown as squares and 95% confidence intervals as error bars. **(c) **Kaplan-Meier breast cancer specific death survival (BCSS) plots are shown for patients with ER+/node negative tumors (n = 130). Panels correspond to the four different age quartiles: 0 to 25%, age 25 to 55 years old patients; 26 to 50%, age 56 to 64 years old patients; 51 to 75%, age 65 to 71 years olds; 76 to 100%, age 72 to 82 years old patients. *P *values correspond to Mantel-Cox log-rank test. SRAP = steroid receptor RNA activator protein.

In multivariate regression analyses of the whole cohort, age, ER status and nodal status were, as expected, associated with poor prognosis (Table 2). SRAP, however, did not act as a proportional predictor in either BCSS or RFS analysis. In the ER-negative group, neither SRAP nor any interaction was a predictor of survival. In the ER-positive group (n = 271), High SRAP was associated with a higher rate of death due to breast cancer (hazard ratio (HR) = 4.213, *P *= 0.007) and higher rate of recurrence (HR = 3.392, *P *= 0.005). In this subgroup, interaction between SRAP and age approached and reached significance, when considering BCSS and RFS, respectively (*P *= 0.060 and *P *= 0.030). Looked at in isolation, the interaction becomes clear (Figure 8). Within the ER-positive subgroup, SRAP is as a poor prognostic marker for BCSS and RFS in the subset of node-negative and young breast cancer patients (n = 60, HR = 8.616, *P *= 0.006 and HR = 3.566, *P *= 0.011, respectively), but not in older or node positive patients (Figures 8a, b).

**Table 2:** Cox multivariate analysis of breast cancer specific and recurrence-free survivals

		Breast cancer specific survival			Recurrence-free survival		
			
Cases	Predictor	Haz Rat	95% CI	*P*	Haz Rat	95% CI	*P*
	**SRAP**	1.580	0.35-7.01	0.547	0.356	0.29-5.63	0.729
**Whole cohort**	**Age**	2.576	1.50-4.40	**< 0.001**	1.507	1.39-3.60	**< 0.0001**
n = 372	**ER**	0.213	0.12-0.36	**< 0.001**	0.124	0.24-0.64	**< 0.0001**
	**Node**	3.214	1.8-5.61	**< 0.001**	1.84	1.67-4.39	**< 0.0001**

	**SRAP**	1.489	0.31-6.99	0.614	1.387	0.29-6.43	0.676
**ER-**	**Age**	2.556	1.30-5.00	**0.006**	2.515	1.29-4.88	**0.007**
n = 101	**Node**	2.788	1.33-5.82	**0.006**	2.547	1.25-5.18	**0.010**
	**SRAP × age**	0.216	0.01-2.61	0.228	0.231	0.01-2.79	0.249
	**SRAP × node**	1.421	0.31-6.99	0.688	1.422	0.29-6.43	0.686

	**SRAP**	4.213	1.47-12.0	**0.007**	3.392	1.46-7.87	**0.005**
**ER+**	**Age**	1.637	0.75-3.54	0.210	1.656	0.88-3.09	0.113
n = 271	**Node**	3.466	1.45-8.26	**0.005**	2.721	1.40-5.27	**0.003**
	**SRAP × age**	0.316	0.09-1.05	0.060	0.337	0.12-0.89	**0.030**
	**SRAP × node**	0.474	1.47-12.0	0.216	0.539	1.46-7.87	0.210

CI = confidence interval; ER = estrogen receptor; Haz Rat = hazard ratio; SRAP = steroid receptor RNA activator protein; + = positive; - = negative.

*P *values correspond to cox regression model. Significant *P *values are bolded.

To further establish the different prognostic value of high SRAP levels in different age subgroups, patients with ER-positive/node-negative tumors (n = 130) have been grouped according to their age (corresponding to quartiles: 0 to 25%, 26 to 50%, 51 to 75%, 76 to 100%). Patients whose age fell in the first and second quartiles (24 to 55 and 56 to 64 years old), but not older patients, had a significant decrease in survival when their primary tumors expressed high SRAP levels (Figure 8c).

## Discussion

In the present study, we have established that several SRAP-like peptides were detectable in breast tumor tissues. Interestingly, in ER positive, and more predominantly within node negative and younger patients, higher levels of the corresponding signal were associated with poor survival.

No data reporting specific detection of SRAP employing the antibody used in our experiments (cat # A300-743A) has been published. It was therefore critical to first validate the use of this antibody for western blot and immunohistochemistry analyses. A doublet migrating at the apparent size of about 30 kDa is detected by western blot in MCF-7 as well as in multiple other cell lines (Figure 1 and data not shown). This size corresponds to the size previously observed using different SRAP antibodies [15,17,19]. The signal decreases upon peptide neutralization and during SRA RNAi experiments. An additional band, also recognized by anti-V5 antibody (data not shown), appears when cells are transfected with a construct encoding an exogenous V5-tagged SRAP. Altogether, these observations strongly suggest that 743A antibody specifically recognizes SRAP.

The endogenous SRAP signal is detected mainly in the cytoplasm of cancer cells *in vitro*. In some cases, a speckled staining can also be seen in the nucleus. This is in agreement with both localizations previously observed for exogenous tagged SRAP [16]. This also corroborates data from Jung and colleagues [21], who co-immunoprecipitated endogenous SRAP from both nuclear and cytoplasmic protein extracts from Hela cells [10,21].

Western blot analysis of breast tumors revealed the presence in some tumors of two additional bands migrating at an apparent size of about 25 kDa and about 40 kDa. Both signals disappear when the antibody is pre-incubated with the corresponding blocking peptide. This suggests that as well as 30 kDa SRAP, these proteins are also specifically recognized by 743A antibody. We have already reported the detection, within some breast tumors, of both 30 kDa and 25 kDa migrating bands [15]. The antibody used was, in contrast to 743A, targeting the N-terminal extremity of SRAP, because it was raised against SRAP amino acids 20 to 34. No bands migrating at the apparent size of 40 kDa had been previously observed. It should however be noted that when blots are overexposed, the 40 kDa band is detectable in MCF-7 cells but not in Hela cells (Figure 3), suggesting potential cell/tissue specificity in the mechanisms responsible for the generation of the corresponding peptide. This raises the possibility that this particular 'higher mass' or 'larger' peptide might be missing SRAP N-terminal extremity but contain additional unknown sequences. The identity of all these SRAP-like peptides remains to be determined. It should however be stressed that several SRA splice variants have now been characterized [10,13,17]. Proteins potentially encoded by some of these alternatively spliced transcripts are missing SRAP N-terminal extremity but still contain SRAP amino acids 180 to 236 recognized by 743A antibody. For example, Ensembl transcript [Ensembl: ENSESTT00000035630] containing intron-1, is expected to encode for a peptide [Ensembl: ENSESTP00000035630] corresponding to the last 162 amino acids of SRAP and with a predicted molecular mass of 18 kDa. As well, other alternative splicing events, such as intron-1 retention conjugated with exon-3 deletion [17], predicts a 220 amino acid residue protein (molecular weight 24.68 kDa) identical to SRAP in its N- and C-terminal extremity but differing in the central sequence.

Further studies are urgently needed to establish the nature of the different splicing events potentially involved in the generation of the different SRA transcripts and their potential protein products. This is critical if we consider that SRA was originally described as a functional RNA whose activity depended on the integrity of the core sequence determined by exon-2 to exon-5 [29]. It has recently been shown that the balance between fully spliced SRA and transcripts still containing intron-1 varied between breast tumors and characterized particular tumor subgroups [30]. Interestingly, alteration of this balance led to a change in breast cancer cell growth [30]. Taken together with size differences between SRA transcripts detectable by Northern blot in breast cancer cells and cells from other origins [7], this suggests that alternative splicing events, leading to different SRA RNAs and potentially to different SRAP-like proteins, might participate in the development of different breast cancer cell phenotypes.

Alternative post-translational modifications might also be responsible for the apparent differences in SRAP molecular mass. For example, a lysine residue at position 21 of the SRAP human sequence is conserved in all vertebrates and is predicted to be sumoylated (data not shown). Sumoylation of this residue could potentially participate to the shift in migration from 30 kDa to 40 kDa [31,32]. Further studies are warranted to address this possibility.

Although only six normal breast cases were analyzed, they all expressed only 30 kDa SRAP at a level lower than that seen in most tumors. This is in agreement with the previous observation that SRA RNA is overall expressed at lower levels in normal compared with matched adjacent tumor tissue [14]. This also raises the possibility that mechanisms responsible for the generation of the 25 kDa and 40 kDa SRAP-like peptides are less frequent in normal than in tumor cells.

Using 743A antibody, significant higher levels of SRAP are found in ER-positive tumors, in PR-positive tumors, and in lower grade tumors. This fits with the pattern of expression of a known activator of steroid receptor, the steroid receptor co-activator 1 (SRC1). Indeed, high SRC1 protein levels have recently been shown to positively correlate with ER, PR levels and low grade in a large cohort of 426 breast cancer patients [33]. In their study, Green and colleagues reported that patients whose tumors expressed higher SRC1 levels had an overall longer survival and a lower recurrence rate [33]. In contrast, we found that high SRAP expression is a strong indicator of poor survival in ER-positive patients. It should be noted that a similar finding was obtained when using a training set, validation set method and X-tile software [34] (data not shown).

All 271 women whose tumors were ER positive had been treated with tamoxifen following surgery. Further analyses are needed to establish whether SRAP should be considered as a prognostic factor, a predictive factor, or both.

Interestingly, the potential indicator power of SRAP appears limited to younger patients whose tumors are ER positive and node negative. It should be stressed that the age cut-point of 64 years (corresponding to median age at time of surgery) has been chosen to divide the cohorts in subgroups with comparable number of cases. A cut-point of 51 years, average age for spontaneous menopause [35,36], would have been more informative to establish the potential link between menopausal status and SRAP prognostic value. The number of cases younger than 51 was, however, too low to perform meaningful statistical analyses. For example, only 21 out of the 271 ER-positive patients were younger than 51 years. Menopause age follows a Gaussian distribution ranging roughly from 40 to 60 years [35,36]. As patients' menopausal status is not registered in the Manitoba Breast Tumor Database, women younger than 51 years could also potentially be menopaused. It is therefore not possible to extrapolate whether SRAP prognostic value is directly linked to menopausal status. Further studies, performed on a cohort consisting of comparable numbers of known pre- and post-menopausal women should be initiated to clarify this issue.

The particular group of younger patients identified in this cohort (i.e ER positive, node negative) usually benefits from better prognosis and is considered as having an increased likelihood of responding to tamoxifen therapy. Our data, however, suggest that among this group, women with higher levels of SRAP might need additional treatment compared with women with lower SRAP levels.

We have previously reported that the detection in breast tumor extracts of 30 kDa SRAP by western blot, but not 25 kDa SRAP, might correspond to a better outcome for ER-positive/node-negative patients [17]. There is therefore an apparent disagreement with the present TMA results that show that higher SRAP is an indicator of shorter survival in ER-positive/node-negative patients. This apparent divergence could be due a variety of reasons. It may potentially result from differences in the cohorts studied. Our previous cohort was indeed smaller (n = 74 versus n = 130), the median ER levels was higher (45.5 fmol/mg versus 37 fmol/mg), the number of events was different (n = 7 deaths versus n = 26) and patients' ages were not available. Further, in the previous western blot analysis, which was performed as outlined earlier with an antibody recognizing the N-terminal region of SRAP, 30 kDa SRAP was detected in only 24 of 74 cases [17]. Using 743A, we have in the present study detected SRAP signal in all but 2 out of 130 ER-positive/node-negative patients. It should also be stressed that, as mentioned above, SRAP staining assessed in the present TMAs corresponds to multiple SRAP-like peptides (25 kDa, 30 kDa and 40 kDa SRAP) whereas only the 30 kDa SRAP was considered in the first study. This also raises the possibility that different peptides might have different prognostic or predictive values.

## Conclusions

Altogether, we have found that several SRAP-like peptides are expressed in breast tumors and that their detection by immunohistochemistry could be used as a new prognostic/predictive marker in younger patients with ER-positive/node-negative breast cancer. Additional studies, performed with other antibodies are warranted to confirm this observation and further establish whether specific SRAP-like peptides have additional predictor values.

## Abbreviations

BCSS: Breast Cancer Specific Survival; DMEM: Dulbecco's modified Eagle's medium; ER: estrogen receptor alpha; FBS: fetal bovine serum; HER: human epidermal growth factor receptor; HR: hazard ratio; LBA: ligand binding assay; PBS: phosphate-buffered saline; PR: progesterone receptor; RFS: recurrence-free survival; SRA: steroid receptor RNA activator; SRAP: steroid receptor RNA activator protein; SRC1: steroid receptor co-activator 1; TMA: tissue micro-arrays.

## Competing interests

The authors declare that they have no competing interests.

## Authors' contributions

YY and GPS equally contributed to this work by performing antibody validation and TMA. CP scored the slides. ZN performed the statistical analysis. SC-K, CC and AB performed western blot analysis and immunofluorescent experiments. PHW, YM, LCM and EL conceived, designed and coordinated the study. All authors drafted, criticized and approved the manuscript.
